# Prehabilitation Utilization for Elective Lower Extremity Joint Replacement Among Older Medicare Beneficiaries

**DOI:** 10.1093/geroni/igaf122.2031

**Published:** 2025-12-31

**Authors:** Jenni Reiff, Jennifer Wolff, John McGready, Jennifer Schrack, Tracy Mroz, Lisa Reider

**Affiliations:** Veteran’s Administration (Atlanta), Decatur, Georgia, United States; Johns Hopkins University - School of Public Health, Baltimore, Maryland, United States; Johns Hopkins Bloomberg School of Public Health, Baltimore, Maryland, United States; Johns Hopkins Bloomberg School of Public Health, Baltimore, Maryland, United States; University of Washington School of Medicine, Seattle, Washington, United States; Johns Hopkins Bloomberg School of Public Health, Baltimore, Maryland, United States

## Abstract

This retrospective cohort study presents the first national characterization of preoperative rehabilitation (“prehabilitation”) for lower extremity joint replacement among older Medicare Fee-for-Service beneficiaries. Using claims data from a 20% sample of Medicare beneficiaries, we included beneficiaries ages 65 and older undergoing incident elective inpatient hip or knee replacement between January 1, 2016, and December 31, 2019, and identified those receiving physical or occupational therapy services initiated within 30 days preceding surgery. Using statistical testing and multivariate linear regression, we estimated utilization trends and characteristics associated with 1) individual receipt of prehabilitation, 2) inpatient facility delivery of prehabilitation, and 3) prehabilitation services (timing, volume, and setting). Of 196,690 included older adult Medicare beneficiaries (mean age 73, 45.4% non-frail, 37.5% hip surgery), 18,739 (9.53%) received prehabilitation. Prehabilitation prevalence increased from 8.03% in 2016 to 11.14% in 2019. Stratifying by operative joint and frailty status, greatest increase was noted among non-frail knee replacement patients (+3.64% between 2016 and 2019). Most prehabilitation patients (77%) received a single visit, primarily in the outpatient physical therapy setting. Likelihood of receiving prehabilitation varied by patient race, gender, financial and health characteristics. More than half (55.7%) of inpatient facilities delivered prehabilitation to at least one patient during the study period; likelihood of delivering prehabilitation varied by facility location, size, and financial characteristics. Findings demonstrate growth in prehabilitation utilization over time, supporting further investigation of why and how providers are implementing prehabilitation, what factors impact patient access, and what strategies may maximize the utility of this service delivery innovation.

